# A Batch of Dentists

**Published:** 1879-05

**Authors:** 


*A Batch of Dentists.*—The Commencements of the various Dental Colleges are over, and the published lists show 233 graduates as the result of the Winter's work. It is earnestly hoped that this large addition to the working force of the country will result in more and better dentistry, and the elevation of the tone of the profession. The Baltimore College turns out 41 graduates, with 82 matriculants; the Ohio College, 24, with 50 matriculants; the Pennsylvania College, 42, with 94 matriculants; the Philadelphia College 49, with 113 matriculants; the New York College 21, with 76 matriculants; the Missouri College 4; the Dental Department of the University of Pennsylvania 25, with 53 matriculants; the Royal College of Ontario 14, with 23 students in attendance.

Nine Colleges. Is it not too many? and cannot the example of Baltimore be taken to heart? Rivalry is a good thing, but there is a point where its value ceases. Fewer schools, the concentration of the energies of those at work in them and for them, and more selective care on the part of those who send students, (for mark it, gentlemen who take office students, the schools take what you send,) and improvement will result. There is need of this last all around. H.

